# A comparison of ClearSight noninvasive cardiac output and pulmonary artery bolus thermodilution cardiac output in cardiac surgery patients

**DOI:** 10.1186/s13741-022-00248-1

**Published:** 2022-06-09

**Authors:** Yuefu Wang, Weiqin Huang, Jiange Han, Yu Tian, Chunrong Wang, Lihuan Li

**Affiliations:** 1 Department of Anesthesiology, Fuwai Hospital, Chinese Academy of Medical Sciences and Peking Union Medical College, Beijing, China; 2 Department of Anaesthesiology and Surgical Critical Care Medicine, Beijing Shijitan Hospital, Capital Medical University, Beijing, China; 3 Department of Anesthesiology, Asian Heart Hospital, Wuhan, Wuhan China; 4 Department of Anesthesiology, Chest Hospital, Tianjin, Tianjin China

**Keywords:** Cardiac output, ClearSight, Noninvasive, Pulmonary artery catheter, Bolus thermodilution, Volume clamp, Finger cuff, Pulse wave analysis

## Abstract

**Background:**

The ClearSight system measures blood pressure non-invasively and determines cardiac output by analyzing the continuous pressure waveform. We performed a multi-center clinical study in China to test the equivalence of cardiac output measured with the ClearSight system (CSCO) and cardiac output measured with the pulmonary artery catheter bolus thermodilution (TDCO) method.

**Methods:**

We included adult patients undergoing cardiac surgery in three Chinese hospitals and measured TDCO and CSCO simultaneously after induction of anesthesia. Hemodynamic stability was required during measurement of TDCO and CSCO. At least four TDCO determinations were performed. The corresponding CSCO was determined as the average over a 30-s period following the injection of each bolus. A data pair for the comparison included the average of three or four accepted TDCO values and the average of the matching CSCO values. Main outcomes included Bland-Altman analysis of bias and standard deviation (SD) and the percentage error (PE).

**Results:**

One hundred twenty-five subjects were enrolled, and 122 TDCO and CSCO data pairs were available for analysis. Ninety-five (75.4%) data pairs were collected in hemodynamically stable conditions, mean (SD) CSCO was 4.21 (0.78) l/min, and mean TDCO was 3.90 (0.67) l/min. Bias was 0.32 (0.51) l/min, and PE was 25.2%. Analyzing all 122 data pairs resulted in a mean CSCO of 4.19 (0.82) l/min and a mean TDCO of 3.83 (0.71) l/min. Resulting bias was 0.36 (0.53) l/min, and PE was 26.4%.

**Conclusions:**

CSCO and TDCO agreed with a low systematic bias. Besides, mean PE was well below the pre-defined 30%. Hemodynamic stability only had a small impact on the analysis. We conclude that CSCO is equivalent to TDCO in cardiac surgery patients.

The trial was retrospectively registered in ClinicalTrials.gov, identifier NCT03807622; January 17, 2019

## Background

Inadequate oxygen supply is one of the main reasons leading to postoperative complications. The objective of perioperative hemodynamic optimization is to improve patient outcomes by maintaining adequate oxygen supply to the tissues. A common approach is to apply a goal-directed therapy (GDT) protocol to titrate fluids, vasopressors, inotropes, and possibly blood products. Often, these protocols require continuous measurement of advanced hemodynamic parameters like cardiac output (CO) and systemic vascular resistance, as well as blood pressure (BP). Many randomized controlled trials and quality improvement projects have demonstrated the utility of GDT to reduce postoperative complications and length of stay in various types of patients and surgical procedures (Kaufmann et al., 2019). Further supported by several meta-analyses (Cecconi et al., 2013; Benes et al., 2014; Michard et al., 2017; Chong et al., 2018; Dushianthan et al., 2020; Giglio et al., 2019), this cumulative evidence has led to the incorporation of GDT principles into several clinical practice guidelines (Mythen et al., 2012; Vallet et al., 2013; Brienza et al., 2019).

Over the past 40 years, we have seen significant progression in the development of technologies to continuously measure advanced hemodynamic parameters. As a result, several invasive methods are available, but not all patients who may benefit from GDT are indicated to receive invasive monitoring. The volume clamp-based technology has been developed as a noninvasive method to continuously measure blood pressure (Penaz, 1975; Wesseling, 1995). This method is implemented in the ClearSight system that uses a finger cuff as the only interface to the patient. Several studies have shown that, compared to invasively measured BP, it is a reliable method to noninvasively measure continuous BP (Martina et al., 2012; de Wilde et al., 2016). Similar to some minimally invasive systems, a pulse contour method is applied to determine continuous cardiac output (Truijen et al., 2012). The noninvasive ClearSight system enables expansion of perioperative hemodynamic optimization to patients who do not routinely receive invasive monitoring yet are at risk to develop postoperative complications.

The gold standard for measuring CO is invasive, pulmonary artery catheter (PAC)-derived cold bolus thermodilution cardiac output (TDCO) (Swan et al., 1970; Ganz et al., 1971). The previous generation of the volume clamp technology has been compared with TDCO in a few small studies (Stover et al., 2009; Bogert et al., 2010; Sokolski et al., 2011; Bubenek-Turconi et al., 2013; Sperna Weiland et al., 2018), and mixed results were reported. As such, the performance to measure CO by the ClearSight system is still debated. Criteria for validation of CO monitors are under discussion, but in general, a new technique has to be accurate and precise compared to a reference method (Cecconi et al., 2009).

The primary objective of this study was to test the equivalence of noninvasive CO measurement with the ClearSight system (CSCO) and PAC-derived TDCO, as determined by a mean bias of 0.5 L/min or less and a percentage error (PE) lower than 30%. This multi-center study was performed in three Chinese hospitals and to our knowledge is the first study to compare CO measured by the ClearSight system against TDCO.

## Methods

This was a prospective, nonrandomized, noninterventional, multicenter trial performed in Chinese patients undergoing open-chest cardiothoracic surgery in accordance with the principles of the Declaration of Helsinki and National Medical Products Administration (NMPA) Good Clinical Practice Decree No. 25 (China, 2016). The study was registered at clinicaltrials.gov (NCT03807622). Ethical approval was obtained from the three participating hospitals in China: Fuwai Cardiovascular Disease Hospital in Beijing, Tianjin Chest Hospital in Tianjin and Wuhan Asia Heart Hospital in Wuhan. All patients provided written informed consent before they participated in the study. Prior to the start of the trial, the research teams were trained on the study protocol, the use of the equipment and performing the measurements.

Adult patients (≥ 18 years old) were recruited from the operating schedule if they were to undergo elective open-chest cardiothoracic surgery and indicated for CO measurements using a PAC catheter. Reasons to exclude patients from the trial were aortic or tricuspid valve regurgitation, aortic valve stenosis, aortic aneurysms, rhythm disorders, intracardiac shunt, or treatment with an intra-aortic balloon pump. Insufficient perfusion of the finger compromises the reliability of the ClearSight measurements. Therefore, we excluded patients with extreme contraction of the smooth muscle in the arteries or arterioles in the lower arm and hand, such as may be present in patients with Raynaud’s disease or Buerger’s disease or with extremely cold hands. Finally, known pregnancy and the inability to place the finger cuff appropriately due to subject anatomy or condition were also reasons to exclude patients.

Noninvasive measurement of BP by the ClearSight system is performed with a finger cuff having a photoplethysmographic system to measure arterial volume and an inflatable bladder to put pressure on the finger arteries. In order to keep the arterial volume constant throughout the cardiac cycle, the cuff pressure has to be adjusted with high frequency. Arterial blood volume can only be kept constant when the cuff pressure continuously matches the arterial BP. As such, the continuous arterial pressure wave can be derived from the cuff pressure (Penaz, 1975; Wesseling, 1995).

The arteries have to be clamped to their “unloaded” volume, i.e., the volume where internal arterial pressure and externally applied pressure are the same. The Physiocal method is used to establish the unloaded volume (Wesseling et al., 1995). It analyzes the sharpness and the curvature of the plethysmogram during short periods of constant cuff pressure. This automatic calibration is repeated regularly because the unloaded volume may change as a function of arterial wall smooth muscle tone. When the measurement remains stable, the Physiocal interval is automatically increased to maximally 70 beats. The measurement is considered stable when the length of the calibration interval is 30 beats or more.

Using a proprietary pulse wave analysis method, beat to beat stroke volume (SV) and CO are determined (Truijen et al., 2012). This method divides the area under the systolic part of the reconstructed BP curve by the aortic input impedance as determined from a 3 element Windkessel model that includes characteristic impedance, arterial compliance and peripheral resistance. For each patient, the model is individualized by using age, gender height, and weight. A more extensive description of this model can be found in the paper by Truijen et al. (Truijen et al., 2012).

A Swan-Ganz pulmonary artery catheter (Edwards Lifesciences, Irvine, USA) connected to a Vigilance II monitor (Edwards Lifesciences, Irvine, USA) was used to measure bolus thermodilution CO. A 10-ml sample of iced glucose solution (5%) was drawn through an iced injectate container (CO-SET+, Edwards Lifesciences, Irvine, USA, or any other container meeting the requirements, as judged by the investigator) and injected in a steady manner within 4 s. Four TD CO determinations, with at least 70 s between 2 injections, were averaged to obtain one single CO value. Each thermodilution curve was visually checked before acceptance. The dilution curve was automatically corrected for the temperature of the blood and of the injectate measured at the entrance of the catheter lumen.

The ClearSight finger cuff was connected to an EV1000 monitor (Edwards Lifesciences, Irvine, USA). Beat to beat hemodynamic data were stored on this monitor as well as markers indicating the exact timing of the thermodilution injections. This enabled determination of the start of a 30-s period where CO measured with ClearSight was averaged. For each patient, a single data pair was collected for the comparison, allowing for a within-group analysis only. A data pair included the average of three or four accepted TDCO values and the average of the matching CSCO values. Measurements were performed after anesthesia induction, but prior to the start of the surgery.

The accuracy of the bolus thermodilution method depends on hemodynamic stability during the measurement (Jansen et al., 1990; Truijen et al., 2018). After the measurement, the following acceptance criteria for each TD CO measurement were applied (Jansen, 1995; Harms et al., 1999; Jansen et al., 2001):

- Mean arterial pressure and heart rate variation was less than 15%
- If one of the four TD CO measurements deviated more than 15% from the average of the four measurements, this CO value was rejected

Similarly, the acceptance criterion for ClearSight data was a Physiocal interval of 30 beats or more.

If less than three hemodynamically stable measurements were available, the results of a patient were not included in the analysis of the primary objective. Calculation of the bias and standard deviation (SD) was performed using the method proposed by Bland and Altman (Bland & Altman, 1986). Percentage error was calculated as twice the SD of the bias divided by the mean of the CO values from TDCO and CSCO. In addition, a linear regression analysis was performed to assess agreement between TDCO and CSCO. Analyses were performed using Excel (Microsoft, Redmond, WA).

The sample size calculation was based on comparing a mean in one-sample equivalence testing (Z-test). In order to capture equivalence between the two CO methods, this study accepted a 90% power, a significance level of 0.05 and a bi-directional 0.5 l/min cutoff. Assuming a true mean bias of 0.0 and a SD of 1.4 l/min, demonstration of equivalence required 87 subjects to reach 90% power for the primary hypothesis. To allow for rejection of data, up to 125 subjects could be enrolled.

## Results

A total of 126 subjects consented to participate in the study. One subject had to undergo emergency surgery leaving 125 subjects enrolled in 3 hospitals: 57 at Fuwai Cardiovascular Disease Hospital, 31 at Tianjin Chest Hospital, and 37 at Wuhan Asia Heart Hospital. In 3 subjects, it was not possible to complete the study procedure resulting in 122 measurement pairs (1 pair from each subject). After applying the acceptance criteria, the data of 27 subjects had to be rejected, resulting in 95 measurement pairs being available for evaluation of the primary endpoint. The primary reason for rejection was hemodynamic instability (23 subjects). In 4 subjects, the calibration interval for ClearSight was less than 30 beats during the measurements.

Baseline demographics and characteristics of the 122 subjects are presented in Table 1. The average subject age was just below 60 years and about three quarters were male. Most (*n* = 108) subjects were classified as ASA III and NYHA II (*n* = 95). The finger cuff was placed on the middle finger of 96% of the subjects. Medium (29%) and large (71%) size cuffs were applied only. No adverse events related to PAC placement or finger cuff use were reported.

**Table 1:** Patient demographics and characteristics

General		
Age [year]		59.81 ± 7.24
Gender M/F		90/32
Body weight [kg]		72.83 ± 12.20
Height [cm]		166.36 ± 8.42
Status		
ASA II/III/IV		11/108/3
NYHA I/II/III		9/95/18
Surgery		
Coronary artery bypass grafting		122
Finger cuff		
Placement	Left/right side Middle finger/ring finger	69/53 117/5
Size medium/large		35/87

*ASA* American Society of Anesthesiologists Physical Status classification, *NYHA* New York Heart Association

**Fig. 1:**
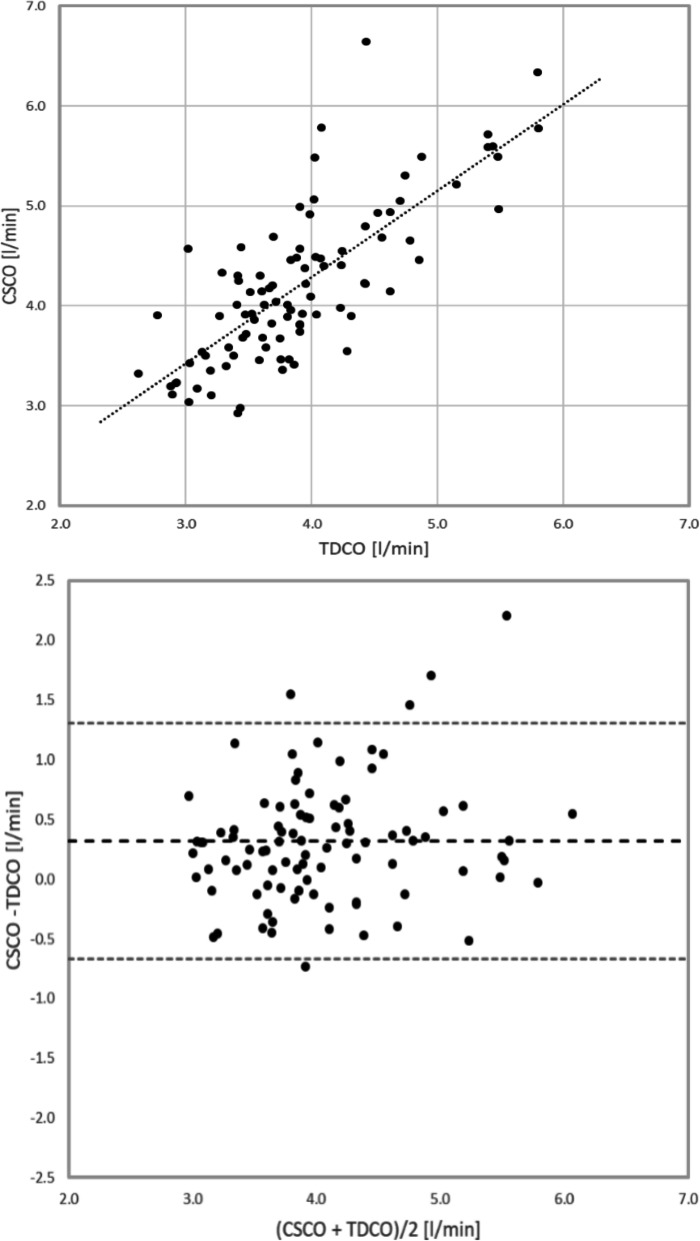
Correlation plot and Bland-Altman plot of hemodynamically stable cases. CSCO, cardiac output measured with the ClearSight system; TDCO, cardiac output measured with the bolus thermodilution method

The primary endpoint was analyzed using the data of 95 subjects. The mean CSCO was 4.21 l/min, and the mean TDCO was 3.90 l/min. The mean bias was 0.32 l/min, with a 95% confidence interval of 0.22–0.42 l/min. The PE was 25.2%. Limits of agreement (LOA) were − 0.67 l/min and 1.31 l/min. Table 2 has further details of the analysis. The correlation coefficient between the TDCO and CSCO was 0.77. The correlation plot with the regression line and the Band-Altman plot with both the bias and the LOA indicated are shown in Fig. 1.

**Table 2:** Cardiac output analysis

	Stable	All
Number of measurement pairs	95	122
CSCO: mean ± SD [l/min]	4.21 ± 0.78	4.19 ± 0.82
Range [l/min]	2.95–6.65	2.61–6.69
TDCO: mean ± SD [l/min]	3.90 ± 0.67	3.83 ± 0.71
Range [l/min]	2.63–5.70	2.25–5.80
Bias: mean ± SD [l/min]	0.32 ± 0.51	0.36 ± 0.53
95% confidence interval [l/min]	0.22–0.42	0.27–0.46
LOA [l/min]	− 0.67–1.31	− 0.68–1.40
PE [%]	25.15	26.43

*CSCO* cardiac output measured with the ClearSight system, *TDCO* cardiac output measured with the bolus thermodilution method, *SD* standard deviation, *LOA* limits of agreement, *PE* percentage error

In addition to the pre-specified analysis using only the data that was measured under hemodynamically stable conditions, we also analyzed the data of all 122 subjects. The mean CSCO was 4.19 l/min, the mean TDCO was 3.83 l/min. The bias was 0.36 l/min and the PE was 26.4%. LOA were slightly wider at -0.68 l/min and 1.40 l/min.

## Discussion

This study demonstrates that CO measured with the ClearSight system and with the pulmonary artery bolus thermodilution method correlate well. Using the Bland and Altman analysis, it was found that the ClearSight system is accurate, i.e., able to measure CO with a low bias of 0.32 l/min. In addition, the precision of the ClearSight system was also within prespecified limits (Critchley & Critchley, 1999), as demonstrated by a PE of 25.15%. We therefore conclude that the ClearSight system meets the criteria for equivalence with the bolus thermodilution method to measure CO.

Uniformly accepted criteria do not exist, but in general, a CO measurement is expected to be accurate and precise (Cecconi et al., 2009; Critchley & Critchley, 1999). In this study, a bias of 0.5 L/min or less was considered clinically acceptable. In addition, the recommended acceptance criterion for PE is 30% (Critchley & Critchley, 1999), although this was heavily debated by Peyton et al. (Peyton & Chong, 2010). In their meta-analysis of forty-seven studies, they reviewed four noninvasive technologies to measure CO. They found PEs ranging from 41.3 to 44.5%, and as a result, they concluded that the relevance in clinical practice of this arbitrary limit should be reassessed. Still, these criteria for accuracy and precision were both met in this study.

There is extensive evidence that perioperative hemodynamic optimization improves outcomes of patients undergoing moderate to high risk surgeries (Chong et al., 2018; Dushianthan et al., 2020; Giglio et al., 2019). However, it is important to acknowledge that no monitoring tool, no matter how accurate, by itself has improved patient outcome. Hemodynamic monitoring systems are measurement tools and their effects on outcomes are only as good as the subsequent protocolized treatment by providers.

Many treatment protocols for optimization require continuous measurement of blood pressure and flow related parameters like SV or CO. The ability to measure these parameters in a totally noninvasive way provides a solution for those surgical patients that may benefit from hemodynamic optimization, but do not have an indication for continuous invasive monitoring.

Although bolus pulmonary thermodilution is considered the clinical gold standard for measuring CO, the accuracy depends on hemodynamic stability during the measurement (Jansen et al., 1990). In order to obtain the best accuracy, we decided to reject thermodilution results in hemodynamically unstable conditions. The 15% variation in MAP and HR we allowed was wider than the 5% and 10% reported previously (Jansen, 1995; Harms et al., 1999; Jansen et al., 2001). However, using these tight criteria would have resulted in too many datasets being rejected. After visual inspection of the BP waveforms, the authors pragmatically decided that variations up to 15% still provided sufficient accuracy for the thermodilution method. Upon analyzing all data pairs, we found that the application of the stability criteria only had a small effect on the results, i.e., bias and percentage error were only slightly higher.

The bias and PE found in this study are in line with results reported in studies (Stover et al., 2009; Bogert et al., 2010; Sokolski et al., 2011; Bubenek-Turconi et al., 2013; Sperna Weiland et al., 2018) where Nexfin, the previous generation of the noninvasive technology with the same algorithm for CO, was compared with the pulmonary artery bolus thermodilution method to measure CO. The reported biases ranged from 0 to 0.44 l/min whereas the reported PEs ranged from 20 to 38%. The PE in the current study is lower than in 4 of the Nexfin studies and this may be related to the fact that we paid extra attention to the accuracy of the reference method and to the selection of time-synchronous data from the continuous recordings.

This study has several strengths and limitations. The high quality of the reference method, a combination of statistical approaches and the adequate powering of the study can be considered as strengths (Saugel et al., 2020). The first limitation is that we only compared absolute CO values of both methods. The need for hemodynamic stability required the surgeons to wait for several minutes. This waiting time and the time to take the measurements could easily take more than 15 min, and this was expected to take even longer during the surgery. Therefore, it was decided to collect only a single data pair per patient at the expense of the ability to perform a within-patient analysis and to analyze tracking of CO changes, something that is relevant when evaluating the effect of interventions. However, a study performed with Nexfin (Bubenek-Turconi et al., 2013) demonstrated reliable tracking of CO changes resulting from preload-modifying maneuvers in post-cardiac surgery patients with a PAC. Secondly, we did not measure particularly high CO values in this study. Compared to the study by Bogert et al. (Bogert et al., 2010), the highest CO value was almost 2 l/min less (6.7 vs. 8.53 l/min). This may be related to the on average shorter and lighter patients being enrolled in this study (72 vs. 87 kg and 166 vs. 176 cm). The last limitation is that only cardiac surgery patients were enrolled in the study. This is a consequence of the objective to use the pulmonary thermodilution method as the reference. There are only limited opportunities to recruit patients that routinely receive PAC monitoring. Still, the measurement was performed during the pre-pump phase, allowing for transferability of the results to other non-cardiac surgery patients. Also, patients with aortic and tricuspid valve abnormalities were excluded from the study.

## Conclusion

CSCO and TDCO agree with a low systematic bias. Besides, mean PE was well below the pre-defined 30%. Hemodynamic stability only had a small impact on the analysis. We conclude that CSCO is equivalent to TDCO measurement with the pulmonary artery catheter in cardiac surgery patients. The noninvasive ClearSight system enables perioperative hemodynamic optimization of those patients not indicated for invasive monitoring.

## Data Availability

The data supporting the findings of this study are available from the corresponding author on reasonable request with permission of the IRB.

## Abbreviations

ASA: American Society of Anesthesiologists Physical Status classification
BP: Blood pressure
CSCO: Cardiac output measured with the ClearSight system
CO: Cardiac output
GDT: Goal-directed therapy
LOA: Limits of agreement
NYHA: New York Heart Association functional classification
PAC: Pulmonary artery catheter
PE: Percentage error
SD: Standard deviation
SV: Stroke volume
TDCO: Cardiac output measured with the bolus thermodilution method
